# Transmembrane Prostatic Acid Phosphatase (TMPAP) Interacts with Snapin and Deficient Mice Develop Prostate Adenocarcinoma

**DOI:** 10.1371/journal.pone.0073072

**Published:** 2013-09-10

**Authors:** Ileana B. Quintero, Annakaisa M. Herrala, César L. Araujo, Anitta E. Pulkka, Sampsa Hautaniemi, Kristian Ovaska, Evgeny Pryazhnikov, Evgeny Kulesskiy, Maija K. Ruuth, Ylermi Soini, Raija T. Sormunen, Leonard Khirug, Pirkko T. Vihko

**Affiliations:** 1 Department of Clinical Chemistry, University of Helsinki and Helsinki University Hospital Laboratory, Helsinki, Finland; 2 Research Programs Unit, Genome-scale Biology & Institute of Biomedicine, University of Helsinki, Helsinki, Finland; 3 Neuroscience Center, University of Helsinki, Helsinki, Finland; 4 Institute of Clinical Medicine, Department of Pathology and Forensic Medicine, School of Medicine, University of Eastern Finland, Cancer Center of Eastern Finland, Kuopio, Finland; 5 Biocenter Oulu and Department of Pathology, University of Oulu, Oulu, Finland; University of Central Florida, United States of America

## Abstract

The molecular mechanisms underlying prostate carcinogenesis are poorly understood. Prostatic acid phosphatase (PAP), a prostatic epithelial secretion marker, has been linked to prostate cancer since the 1930's. However, the contribution of PAP to the disease remains controversial. We have previously cloned and described two isoforms of this protein, a secretory (sPAP) and a transmembrane type-I (TMPAP). The goal in this work was to understand the physiological function of TMPAP in the prostate. We conducted histological, ultra-structural and genome-wide analyses of the prostate of our PAP-deficient mouse model (PAP^−/−^) with C57BL/6J background. The PAP^−/−^ mouse prostate showed the development of slow-growing non-metastatic prostate adenocarcinoma. In order to find out the mechanism behind, we identified PAP-interacting proteins byyeast two-hybrid assays and a clear result was obtained for the interaction of PAP with snapin, a SNARE-associated protein which binds Snap25 facilitating the vesicular membrane fusion process. We confirmed this interaction by co-localization studies in TMPAP-transfected LNCaP cells (TMPAP/LNCaP cells) and *in vivo* FRET analyses in transient transfected LNCaP cells. The differential gene expression analyses revealed the dysregulation of the same genes known to be related to synaptic vesicular traffic. Both TMPAP and snapin were detected in isolated exosomes. Our results suggest that TMPAP is involved in endo-/exocytosis and disturbed vesicular traffic is a hallmark of prostate adenocarcinoma.

## Introduction

The association between prostate cancer and serum prostatic acid phosphatase (PAP; *ACPP*; EC 3.1.3.2) has been known for more than 70 years [1]. Nevertheless, the molecular mechanisms underlying this association are still poorly understood. In spite of this, the connection between secreted PAP and prostate cancer contributed to the development of Sipuleucel-T, the first FDA-approved vaccine for cancer therapy targeting PAP-expressing cells [2] even when in advanced/androgen-independent prostate cancer tissue, the expression of PAP is down-regulated [3]. Therefore, our goal is to elucidate the pathways where PAP and in particular its transmembrane isoform is involved.

PAP is a histidine acid phosphatase [4] from which two isoforms have been cloned, the secreted (sPAP) and the transmembrane type-I (TMPAP). Both are splice-variants of the same gene and widely expressed in different tissues, in both sexes [5]. The current evidence does not support the existence of a third, cytosolic cellular form of PAP, as it has been suggested in the literature but never cloned [6]–[8]. Topologically, TMPAP contains an N-terminal phosphatase activity domain which is extracellular when TMPAP is in the plasma membrane and intra-luminal when it is trafficking in vesicles, and a C-terminal domain with a cytosolic tyrosine-based endosomal-lysosomal (including MVE) targeting signal motif (YxxΦ) [5]. TMPAP also co-localizes with flotillin and LAMP2 [5], which are known markers for exosomes [9], [10].

The prostate gland is fundamentally a secretory organ, and it is known that the secretion of specialized exosomes (prostasomes) is essential for the maintenance of the spermatozoa [11]. Exosomes are nanovesicles originated from multivesicular endosomes (MVE), which contain protein, lipid, DNA, RNA and/or microRNA molecules [12]. Also, it has been shown that exosomes are involved in the promotion of cancer cell proliferation and survival [13], and an increased level of prostasomes (exosomes) has been detected in plasma of prostate cancer patients [14].

PAP exerts its phosphatase activity *in vitro* against β-glycerophosphate [15], lysophosphatidic acid [16], and phosphoamino acids [17] and has 5′-nucleotidase activity [18]. *In vivo*, the ecto-5′-nucleotidase activity of PAP is responsible of dephosphorylating adenosine monophosphate (AMP) to adenosine [18], [19] leading to the activation of A1-adenosine receptors in the dorsal root ganglia (DRG) [19]. PAP regulates the levels of adenosine and phosphatidylinositol 4,5-bisphosphate [PI (4,5) P_2_], an essential regulator of vesicular traffic [20], reducingsensitivity to painful stimuli [19], [21].

SNARE proteins comprise a large family found in yeast and mammalian cells, with the primary function to mediate docking and fusion of vesicles with the cell membranes [22] in regulated endo-/exocytosis [23]. Snapin is a SNARE-associated protein [24] interacting with Snap25, Snap23 or Snap29, and increasing the binding of the calcium sensor synaptotagmin to the SNARE complex [25]. Snapin also forms part of the BLOC1 protein complex, which is necessary for the biogenesis of vesicles in the endosomal-lysosomal pathway [26]. Increasing evidence shows that snapin is important in retrograde axonal transport, late endosomal-lysosomal trafficking and glucose-induced insulin exocytosis. In mediating retrograde axonal transport, snapin acts as a dynein adaptor protein for BDNF-TrkB (brain-derived neurotrophic factor – tyrosine kinase receptor B) activated signaling complexes. This interaction leads to the delivery of TrkB signaling endosomes from axonal terminals to cell bodies, which is an essential mechanism for dendritic growth of cortical neurons [27]. Moreover, snapin deficiency in neurons leads also to accumulation of immature lysosomes due to impaired delivery of cargo proteins from late endosomes to lysosomes [28]. In addition, snapin as a target of protein kinase A (PKA), was found to be a critical regulator of glucose-stimulated insulin exocytosis in pancreatic β-cells by promoting the interaction and assembly of insulin secretory vesicle-associated proteins Snap25, collectrin and Epac2 [29].

The mouse prostate consists of three different lobes: anterior (AP), dorsolateral (DLP) and ventral prostate (VP); and it does not show spontaneous development of neoplasia [30]. The mouse prostate lobes have characteristic histology which has been described previously [31], [32]. Briefly, all prostate lobes show a monolayer epithelium with eosinophilic columnar cells and eosinophilic secretion which is paler in VP than in AP and DLP. Each ductin the lobes is surrounded by a thin fibromuscular sheet composed mainly of smooth muscle cells and collagen fibers. In the AP epithelium, the cell nucleus is central and the epithelium has a high number of infoldings and papillary structures. The DLP epithelium has central to basal nucleus and a moderate degree of infolding. The VP epithelium is characterized by basal nucleus and focal infoldings.

To understand the physiological function of PAP, we studied the prostate of our PAP-deficient mouse model (PAP^−/−^) [33]. The PAP^−/−^ mouse prostate showed disturbed vesicular trafficking, loss of cell polarity and development of slow-growing non-metastatic prostate adenocarcinoma. Here we report the interaction of TMPAP with snapin; and suggest that TMPAP regulates endo-/exocytosis and the disruption of these processesis a hallmark of prostate adenocarcinoma.

## Materials and Methods

### Ethics statement

The animal protocols were approved by the Animal Experimentation Committee of the University of Oulu and ELLA – The National Animal Experiment Board of Finland. The project license numbers are 044/11 and STH705A/ESLH-2009–08353/Ym-2.

### Mice

Mice deficient in PAP were generated by replacing exon 3 (*ACPP* ^Δ3/Δ3^) of the prostatic acid phosphatase gene (*ACPP, PAP*) with the neo gene as described earlier [33] thereby abolishing the expression of both PAP isoforms. The fertility status in the PAP^−/−^ mice was not affected by the gene modification. PAP^−/−^ mice were backcrossed to the C57BL/6J strain (Harlan Laboratories Inc.) for 16 generations to obtain homogenous background. Age-matched C57BL/6J male mice were used as controls in all the experiments.

### Transmission electron microscopy

DLP samples from age-matched PAP^−/−^ and PAP^+/+^ mice were fixed in a mixture of 1% glutaraldehyde and 4% formaldehyde in 0.1 M phosphate buffer for TEM. The samples were post-fixed in 1% osmium tetroxide, dehydrated in acetone, embedded in Epon Embed 812 (Electron Microscopy Sciences) and analyzed at the Biocenter Oulu EM core facility using Philips 100 CM Transmission Electron Microscope with CCD camera.

### Yeast two-hybrid analysis

To screen for interacting partners of human TMPAP, yeast two-hybrid screening was performed using the Matchmaker Gal4 two-hybrid System 3 (Clontech) in accordance with the manufacturer's instructions. The bait construct consisted of the coding region of human TMPAP (GeneBank accession BC007460, nucleotides 51–1304, except the starting methionine was changed to valine) cloned in frame into NcoI/SmaI sites of pGBKT7 using PCR generated linkers. A human thymus cDNA library cloned in pACT2 (Clontech) was used as the prey. The bait and prey plasmids were co-transformed into *Saccharomyces cerevisiae Mav* 203 strain according to the Clontech's two-hybrid protocols. Inserts of positive clones were amplified by PCR, and the DNA was automatically sequenced.

### The Föster resonance energy transfer (FRET) analysis

The FRET variant acceptor photobleaching was used. In this technique, the efficiency of energy transfer between two molecules (and consequently the interaction between them) is measured by comparing the fluorescence of the donor molecule before and after the selective photobleaching of the acceptor moleclule [34]. Human TMPAP-GFP and the control GFP plasmid constructs have been previously described [5]. Human snapin (NM_012437, nt 76–486) was cloned into pDsRed-Monomer-C1 vector, between SalI/BamHI restriction sites. LNCaP cells were obtained from the American Tissue Culture Collection (ATCC) and cultured in 10% FBS (Promocell), L-Glutamine (2 mM), Penicillin (100 U/ml), Streptomycin (100 µg/ml), RPMI-1640 (Sigma) on polylysine-coated petridishes (Mattek). Cells were transfected using Lipofectamine 2000 (Invitrogen) according to manufacturer's instructions. In our experiments we used GFP and DsRed fluorophores as donor and acceptor, respectively, and co-expressed TMPAP-GFP and snapin-DsRed plasmids in LNCaP cells. GFP/DsRed FRET pair provides excellent wavelength separation of donor and acceptor spectra. For FRET positive control we used a DNA construct produced by the connection of EGFP and DsRed sequences with a 23–amino acid linker. Co-transfected pEGFP-C1 and pDsRed-Monomer-C1 vectors have been used as negative controls.

Cultured cells were mounted 24 hours after transfection and epifluorescent images were acquired using an Olympus CellR imaging system with 60× oil immersion NA 1.45 objective. Images were collected with a CCD camera (Orca, Hamamatsu). The system was equipped with automated filter wheels for excitation filters and emission beam-splitter/emission-filter cubes for epifluorescence imaging. GFP fluorescence was excited at 450 nm and collected at 510/40 nm. DsRed fluorescence was excited at 575 nm and collected at 640/50 nm. Acceptor fluorescence was bleached for 5 minutes with maximal burner power.

Images were quantified and processed using Olympus Biosystems AnalySIS software, ImageJ (freely available at http://rsb.info.nih.gov/ij/) and ImagePro 5.1 (Media Cybernetics). Background fluorescence was subtracted prior to calculations. The FRET efficacy defined as the percentage of donor fluorescence increase was calculated with the following equation: E = 1-(Ib/Ia), where ‘Ib’ is the fluorescence intensity of the donor before photobleaching and ‘Ia’ is the post-bleach fluorescence intensity.

### Histology, immunohistochemistry, proliferation and apoptosis analyses, microarray analyses, generation of stable transfected LNCaP cells, immunofluorescence and co-localization studies, comparative genomic hybridization (CGH), isolation of exosomes and Western blot analyses

The detailed methodology can be found in the Supplementary Methodology in File S1.

### Accession codes

Gene expression files containing microarray raw-data can be accessed from ArrayExpress repository database (accession number E-MTAB-1191).

## Results

### PAP-deficiency in mouse prostates leads to development of prostate adenocarcinoma

**Figure 1:**
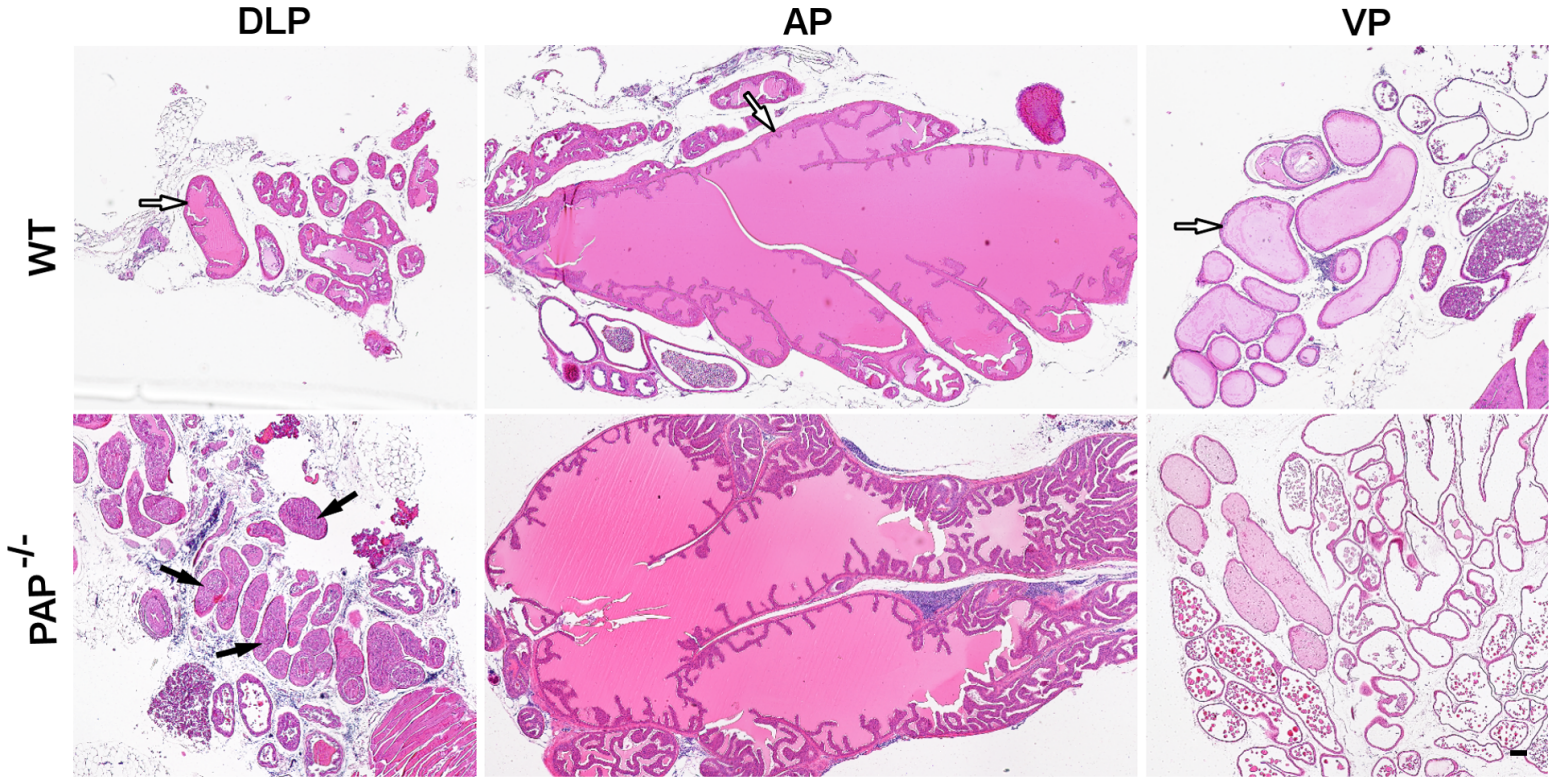
DLP lobeexhibits the primary changes in the PAP^−/−^ mouse prostate. The panels show an overview of the 12-old mice prostate dissected lobes. The DLP, AP and VP lobes were dissected from WT and PAP^−/−^ mouse. The monolayer epithelium (white arrows) is seen in all the lobes of the WT mouse, whereas in the PAP^−/−^ mouse an increased amount of cells is present in the lumen of the DLP lobe (black arrows). The AP and VP of PAP^−/−^ mouse show no significant changes. Scale bars: 100 µm.

**Figure 2:**
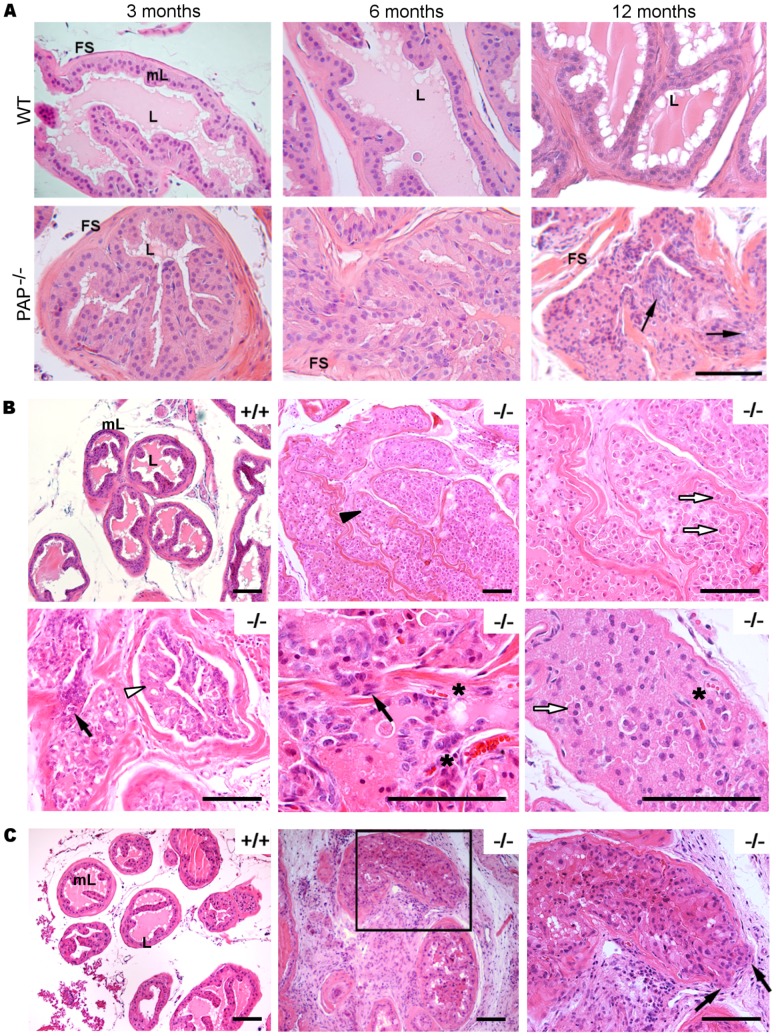
PAP^−/−^ mice develop prostate adenocarcinoma. A, age related histological changes in PAP^−/−^ mouse DLP. Epithelial hyperplasia was present in DLP of 3 month-old PAP^−/−^ mice. The lumen was filled with dysplastic epithelial cells, and mPIN structures were observed in 6 month-old animals. Bulging of epithelial cells into the stroma (black arrows) through loosen fibromuscular sheath and prostate adenocarcinoma were present in 12 month-old mice. Scale bars: 100 µm. (*n* = 8). B, morphological abnormalities present in 12 month-old PAP^−/−^ mouse prostates. Glands were filled with epithelial cells (black arrow head). Dyscohesive cells with double nuclei were present (white arrows), as well as sites of microinvasions of hyperchromatic epithelial cells with prominent nucleoli (black arrow). Cribriform structures (white arrowhead) and blood vessels among neoplastic epithelial cells (*) were also observed. Scale bars: 100 µm. (*n* = 8). C, 24 month-old PAP^−/−^ mouse DLPs. Cells invaded the surrounding stroma and inflammation is present. Microinvasion of cells into the stroma and bulging is clearly observed (black arrows). Scale bars: 100 µm. (*n* = 8). FS: fibromuscular sheath, L: lumen, mL: monolayer epithelium.

PAP-deficiency led to slow development of prostate neoplasia in DLP and AP. The progressive changes in mouse DLP were observed in all the PAP^−/−^mice examined (*n* = 8), detecting a hyperplastic growth already at the age of 3 months, followed by mouse prostatic intraepithelial neoplasia (mPIN) at 6 months and prostate adenocarcinoma at 12 months (Fig. 1 and 2A). The follow-up of the disease in mice spanned until 26 month-old, where the presence of other pathologies arose as strain background or due to mouse aging.

**Figure 3:**
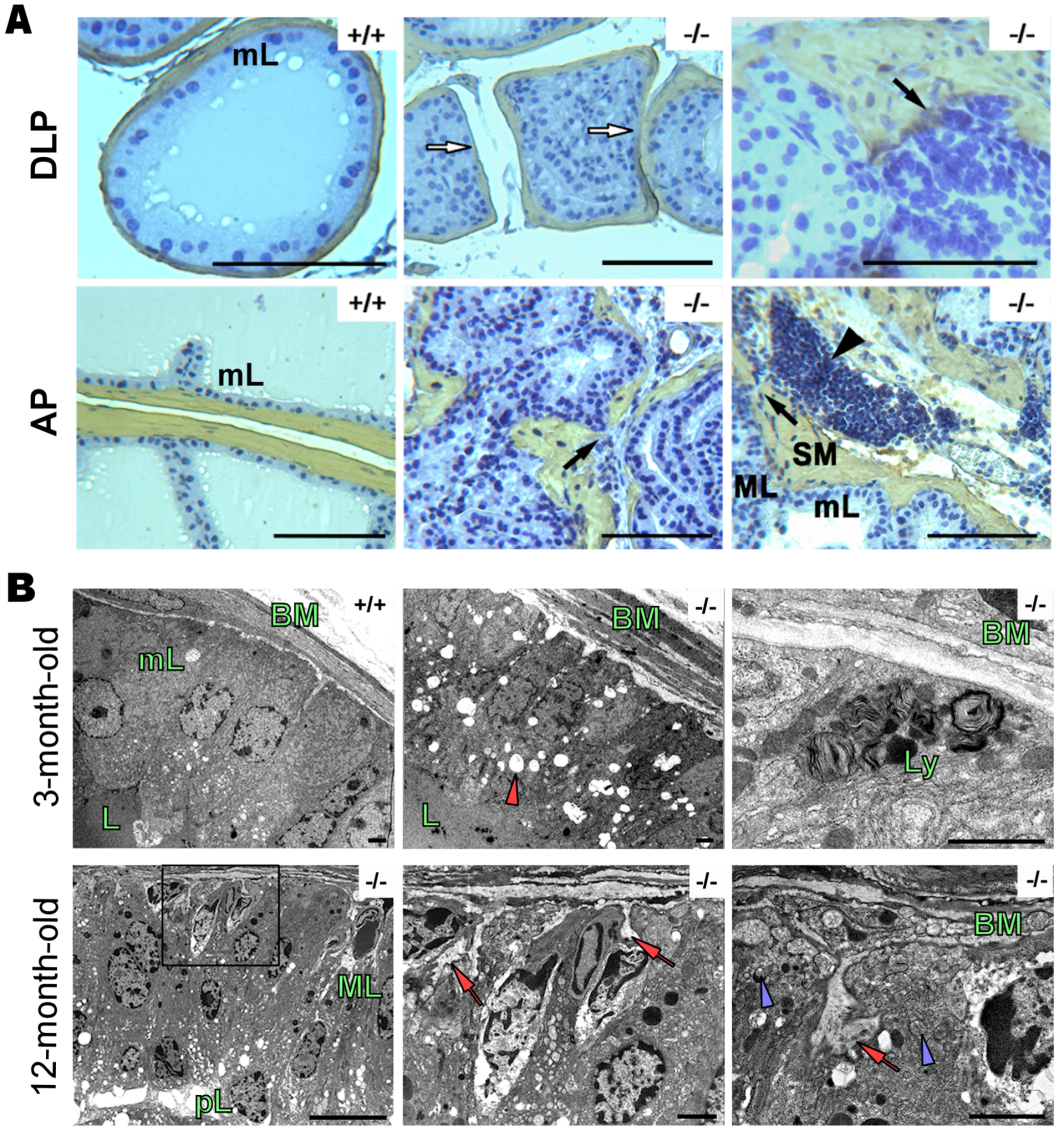
The prostate adenocarcinoma in PAP^−/−^ mice is also detected by immunohistochemistry and electronmicroscopy. A, smooth muscle actin (SMA) immunohistochemistry in 12 month-old mice. Monolayer epithelium (mL) and open lumen in PAP^+/+^ DLP. White arrows show the broken fibromuscular sheath (SM, smooth muscle) and bulging of epithelial cells to the stroma. Prostate adenocarcinoma (black arrows) is present in AP and DLP, showing a multilayer epithelium (ML) and inflammatory cells (black arrowhead) spreading in neighboring areas. Scale bars: 100 µm. (*n* =  4, per group). B, ultrastructural changes in 3 month-old and 12 month-old PAP^−/−^ mouse DLPs. Monolayer epithelium, regular basement membrane (BM) and apical secretion are clearly seen in PAP^+/+^ mouse DLPs. 3 month-old PAP^−/−^ DLPs show irregular BM and numerous apical vacuoles (red arrow head), as well the presence of basal lysosomes (Ly). In 12 month-old PAP−/− mouse DLPs, the epithelium has transformed to a multilayer epithelium containing hyperchromatic nuclei with multiple nucleoli. Pseudolumens (pL) have formed as a result of the growing and fusion of the epithelium. Invaginations of BM (red arrows) into the epithelium and numerous vesicles in the basal side of the cells (blue arrow heads) were additional signs of the transformation in the cells. Scale bars: 2000 nm (*n* = 4, per group).

All the PAP^−/−^mice analyzed at the age of 12 months had developed prostate adenocarcinoma (*n* = 8, Fig. 2B). Pathological acini were filled with non-cohesive pleomorphic epithelial cells, with enlarged hyperchromatic nuclei and prominent nucleoli. The presence of neoplastic acinar cells in the lumen was confirmed with pan cytokeratin staining (Fig. S1 in File S1). The fibrotic stroma surrounding the acini appeared to be invaded by cells, with bulging areas and fusion of acini. Cribriform structures were also observed, additionally to numerous blood vessels among neoplastic epithelial cells. The histological pattern was consistent with locally invasive prostate adenocarcinoma. In the 24 month-old PAP^−/−^ mice, we observed an increasedamount of cells in the AP lumen (Fig. 2A) and a clear invasion of the surrounding areas as well as increased amount of inflammatory cells (*n* = 5, Fig. 3C). However, we did not detect metastatic lesions in other studied organs such as brain, liver, lungs and lymph nodes at any age analyzed.

The breakdown of the fibromuscular sheath and invasion of the epithelial cells into stroma were also detected with smooth muscle β-actin (SMA) staining (Fig. 3A). Bulging of the cells could be seen in atypical acini, as well as adenocarcinoma invasion. Crowding of inflammatory cells was detected in sites of microinvasive adenocarcinoma.

**Figure 4:**
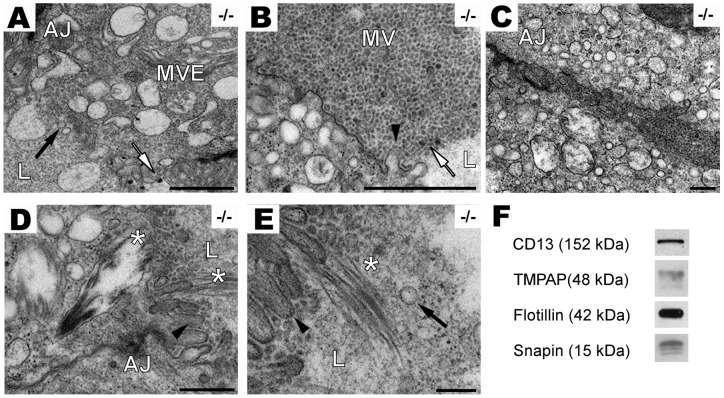
PAP^−/−^ mouse DLPs release exosomal-like microvesicles. A, electronmicroscopy images show the presence of electron-dense (white arrow) and electron-lucent (black arrow) microvesicles (∼30 to 80 nm) in the lumen of the acini, and MVE containing microvesicles in the apical part of the cell. Scale bar: 1,000 nm. B, numerous microvesicles are present in the apical region of PAP^−/−^ DLP and secreted into the lumen, decreased amount of microvilli is observed (black arrowheads) (scale bar: 1,000 nm). C, microvesicles are secreted into basolateral intercellular space of PAP^−/−^ DLP (scale bar: 2000 nm). D, lamellar body-like structures (*) are inside the epithelial cell (scale bar: 500 nm) and E, released into the lumen (*). Scale bar: 200 nm. F, TMPAP and snapin are also present in exosomes. Immunoblots of exosomes isolated from TMPAP/LNCaP cell culture medium. Flotillin and CD13 were used as exosomal and prostasomal marker respectively.

Important changes observed by transmission electron microscopy (TEM) included irregularities and invaginations of the basement membrane into the epithelium (Fig. 3B) in addition to the presence of lysosomes and MVE in the basal side of the cell. The prostatic epithelium of PAP^−/−^ mice lost the regular structure of uniform columnar monolayer transforming to a cuboidal multilayer epithelium with hyperchromatic nuclei and presence of pseudo-lumens as a sign of loss in cell polarity. Further analysis of prostate ultrastructural changes showed an increased number of electron-lucent enlarged vacuoles (Fig. 4A and B), and bursting of luminal exosome-like vesicles of30–80 nm in diameter (Fig. 4B). Exosome-like vesicles in the intercellular space and disintegration of the apical microvilli, indicates also loss of cell polarity (Fig. 4B and C). Lamellar body–like structures were observed in PAP^−/−^ mice DLP cells and their contents secreted into the lumen (Fig. 4D and E).

**Figure 5:**
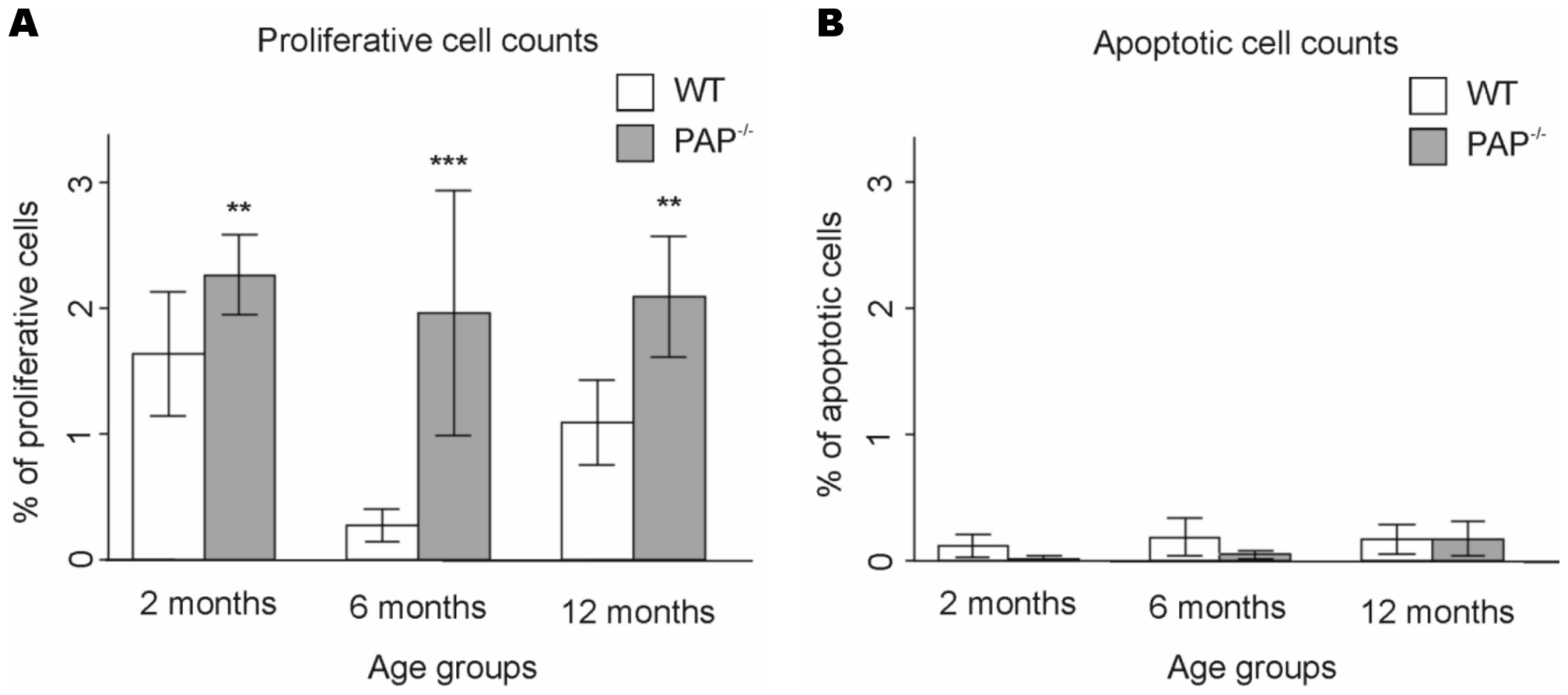
Proliferation of DLP cells is increased in PAP^−/−^ mice compared to WT mice. A, bar plot showing the ratio (as percentage) between proliferative cell counts and total amount of cells. (**, P value <0.01; ***, P value <0.001). Error bars indicate S.E.M. values. B, bar plot showing the ratio (as percentage) between apoptotic cell counts and total amount of cells. Error bars indicate S.E.M. values.

Due to the gradually increased number of cells in the prostate acini, we determined the status of proliferation and apoptosis in the tissue. As a result, the proliferation was significantly increased in the three- (P-value = 4.3×10^−3^, *n* = 4), six- (P-value = 1.3×10^−15^, *n* = 4) and 12 month-old (P-value = 3.9×10^−5^, *n* = 4) PAP^−/−^ mice DLP, but the apoptosis status was not different between genotypes at the same time points (P-values 0.3, 0.1, and 0.9 respectively, *n* = 4) (Fig. 5 and Tables S1 – S4 in File S1).

### PAP interacts with SNARE associated protein, snapin

To determine the effects of PAP deficiency at gene expression level, we performed microarray experiments of mouse prostatic tissue. The Gene Ontology analyses of differentially expressed genes showed, among the most significant groups, those genes associated with regulated secretory pathway, neurotransmitter secretion (*Snap25, Syt1, Syt4, Nrxn1, Cart, Abpa, Cplx1*) and synaptic vesicle traffic (*Rab3c, Syp, Syn2, Syngr1, Syt1, Syt4, Syt10, Svop, Bcan, Timp4*) (Table 1 and Tables S5 and S6 in File S1). Comparative genomic hybridization analysis of DNA prostate samples performed at 6-, 15- and 25 month-old showed no differencies in copy number between the genotypes (wild type C57BL/6J and PAP^−/−^ mice, data not shown).

**Table 1:** Significant ontological terms obtained with GoMiner software from two-color microarrays experiments.

GO ID	*P*-value	Term	Genes
5576	<0.0001	extracellular region	
**7268**	**<0.0001**	**synaptic transmission**	***Nrxn1, Htr3b, Edn1, Timp4, Syn2, Gria1, Tac1, Ncam1, Kif5a, Pnoc, Snap25, Htr3a, Syp, Bcan, Rab3c, Syt10, Cplx1, Gad2, Syt4, Abpa, Grm7, Rasgrf1, Cart, Syt1***
**43005**	**<0.0001**	**neuron projection**	***Gria1, Gria3, Tac1, Ncam1, Mapt, Kif5a, Uchl1, Esr2, Cntn2, Prph1, Nef3, Ttyh1, Sncg, Syt4, Gad2, Nefl, Gap43, Syt1, Kif5c***
**45202**	**<0.0001**	**synapse**	***Nrxn1, Htr3b, Syn2, Thbs4, Gria1, Gria3, Snap25, Htr3a, Nef3, Syp, Bcan, Syngr1, Syt10, Syt4, Gad2, Gabra4, Mgll, Grm7, Syt1***
**30424**	**<0.0001**	**axon**	***Ttyh1, Sncg, Tac1, Gad2, Ncam1, Mapt, Nefl, Gap43, Uchl1, Cntn2, Prph1, Nef3***
**8021**	**<0.0001**	**synaptic vesicle**	***Syngr1, Timp4, Syn2, Syt10, Syt4, Svop, Rab3c, Syp, Bcan, Syt1***
4293	<0.0001	tissue kallikrein activity	
42044	<0.0001	fluid transport	
32501	0.0001	multicellular organismal process	
7267	0.0001	cell-cell signaling	
19226	0.0001	transmission of nerve impulse	*Nrxn1, Timp4, Syn2, Pnoc, Htr3a, Cldn11, Rab3c, Cplx1, Gad2, Syt4, Grm7, Rasgrf1, Edn1, Htr3b, Gria1, Ncam1, Tac1, Kif5a, Snap25, Syp, Bcan, Syt10, Abpa, Cart, Syt1*
3001	0.0001	generation of a signal involved in cell-cell signaling	
45055	0.0001	regulated secretory pathway	*Nrxn1, Timp4, Lat, Syn2, Cplx1, Syt4, Abpa, Snap25, Cart, Rab3c, Syt1*
30672	0.0001	synaptic vesicle membrane	*Syngr1, Syn2, Bcan, Syp, Syt1*
15026	0.0001	coreceptor activity	
5372	0.0001	water transporter activity	
15250	0.0001	water channel activity	
15722	0.0001	canalicular bile acid transport	
5179	0.0002	hormone activity	
7269	0.0002	neurotransmitter secretion	*Nrxn1, Timp4, Syn2, Cplx1, Syt4, Abpa, Snap25, Rab3c, Cart, Syt1*
1772	0.0002	immunological synapse	
6833	0.0003	water transport	
**31226**	**0.0005**	**intrinsic to plasma membrane**	***Nrxn1, Treh, Aqp7, Kcnj5, Aqp9, Aqp5, Cldn11, Cntn2, Htr3a, Cd28, Rhbg, Kcnc1, Lat, V1rc12, Itga9, Acvr1b, Aqp8, Tcrb-V13, Kcnv2, Gabra4, Tpbg, Tyro3, Gp5, Slc12a2, Htr3b, Gria1, Gria3, P2rxl1, Mme, Slco1a6, Abcb11, Snap25, Xtrp3s1, Cnr1, Adcy3, Raet1a, Syngr1, Cd5, V1rg9, Mup1, Ptprj, Slc15a2, Omg, Adora1, Olfr43, Slco1a1***
**45104**	**0.0005**	**intermediate filament cytoskeleton organization and biogenesis**	***Nefl, Krt6b, Prph1, Nef3***
**5883**	**0.0005**	**neurofilament**	***Nefl, Prph1, Nef3***
**30136**	**0.0006**	**clathrin-coated vesicle**	***Syngr1, Timp4, Syn2, Syt10, Syt4, Svop, Ncald, Bcan, Syp, Rab3c, Syt1***
5232	0.0006	serotonin-activated cation-selective channel activity	
5615	0.0007	extracellular space	
48812	0.0007	neurite morphogenesis	
48667	0.0007	neuron morphogenesis during differentiation	
7409	0.0008	axonogenesis	

**GO ID**: gene ontology ID accession number. ***P*** **-value**: *P*-value for the number of changed genes in the input list, significant *P*-value <0.05. **Term**: associated ontological term. **Rows in bold**: relevant ontological groups for vesicular transport. Mice in microarray experiment per group, *n = *3.

The disturbed exocytosis observed in the ultrastructural studies of PAP^−/−^ prostates and the differential expression of genes related to vesicle fusion, such as *Snap25, Syt1, Syt4* and *Cplx1*, led us to search for proteins interacting with TMPAP. The yeast two-hybridization screening of human thymus library detected seven out of 15 clones expressing snapin (NM_012437.3), as a clear candidate for interaction with TMPAP.

**Figure 6:**
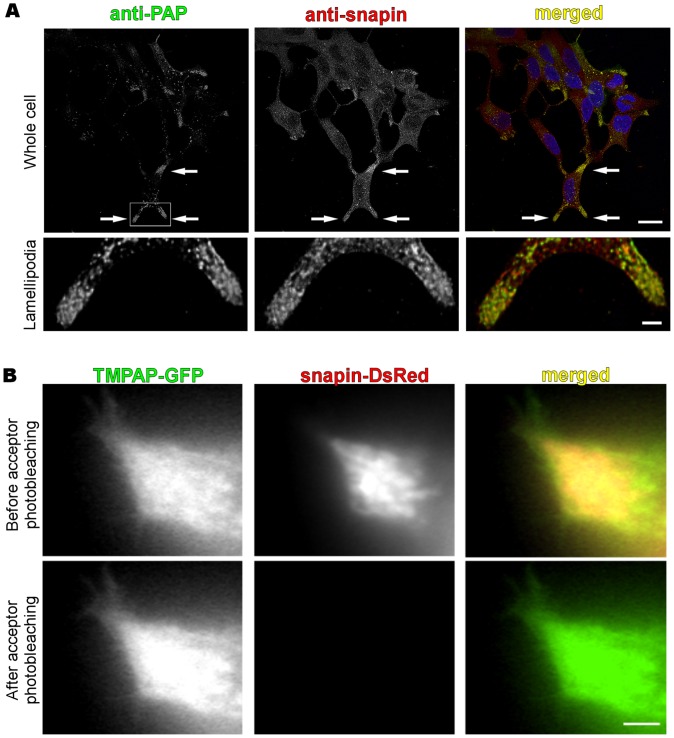
TMPAP co-localize and interact with snapin in the cell lamellipodia. A, co-localization (yellow) of TMPAP (green) with snapin (red) was observed in the vesicles and lamellipodia of the TMPAP/LNCaP cells. Arrows mark the co-localization points in the upper panel (scale bar: 20 µm). Lower panel (scale bar: 3 µm) showing the lamellipodia region, amplification of the area marked with a box in the upper panel (left). B, intensification of donor (TMPAP-GFP) fluorescence in LNCaP cells was observed after acceptor (snapin-DsRed) photobleaching which confirms FRET between two molecules (Scale bar: 2 µm).

To validate the yeast two-hybrid result, double-immunofluorescence staining of PAP and snapin in TMPAP/LNCaP cells showed co-localization of these two proteins in vesicular structures and cell membrane (Fig. 6A). The quantification studies displayed relatively low Pearson's correlation coefficient when the whole cell was analyzed (0.485±0.012). However, when the co-localization was quantified exclusively in the cell lamellipodia the Pearson's correlation coefficient reached a value of 0.680±0.013. This coefficient value not only shows that TMPAP co-localized with snapin but it could imply an interaction between TMPAP and snapin in these cell regions. Therefore, to confirm this hypothesis of interaction, the FRET variant acceptor photobleaching, which gives an *in vivo* proof of the physical protein-protein interaction, was used to determine the interaction between TMPAP and snapin. Data analysis revealed significant FRET between TMPAP and snapin (FRET efficacy 9.3±1%, *n* = 12 cells) compared to experiments with negative control (−0.4±0.7%, *n* = 9 cells, P<0.0001) while in experiments with positive control FRET efficacy reached a level of 37.7±6.5% (Fig. 6B).

Hence our previous results have showed the co-localization of PAP and flotillin, which is a protein also used as exosomal marker [9], wecorroborate by Western blot the presence of PAP inisolated exosomes produced by stable transfected TMPAP/LNCaP cells. The results showed the presence of TMPAP as well as snapin in the exosomal fraction in addition to flotillin and CD13, a prostasomal marker [35] (Fig. 4F).

## Discussion

Prostate cancer is a disease of complex etiology, in which genetic and epigenetic mechanisms are involved. PAP was the first prostate cancer marker, and its usefulness was based in the assessment of its serum activity levels. We have previously shown that in addition to the secretory PAP, a transmembrane isoform is widely expressed in different mouse organs such as prostate, salivary glands, thymus, lung, kidney and brain, amongst others. TMPAP is also present in androgen sensitive prostate cancer cells (LNCaP), but absent in androgen insensitive prostate cancer cells (PC3) [5].

In all the PAP^−/−^ mice, progressive changes were observed in the prostatic tissue leading to the development of prostate adenocarcinoma at the age of 12 months. Despite the presence of prostate cancer, we have not detected any metastatic lesions. Histologically, the mouse DLP has been considered to be the analogous area to the peripheral zone of the human prostate, where the majorities of adenocarcinomas reside [36]. In this regard, Roy-Burman *et al*. suggested that the mouse models carrying genetic modifications that affect the tumor development in the DLP are more significant for the studies on those pathologies associated with the peripheral zone of the human prostate [37].

In humans, it has been observed that PTEN (phosphatase and tensin homolog deleted on chromosome 10) is downregulated in prostate cancer tissue specimens [38]. PTEN antagonize PI3K (phosphoinositol 3-kinase) activity by dephosphorylating phosphoinositol (3,4,5)-triphosphate which is an activator of the AKT pathway leading to cell survival. The PTEN prostate cancer mouse model showed a development of prostate cancer which resembles the stages of the disease in humans, starting with hyperplasia at 4 weeks of age, mPIN, prostate microinvasive adenocarcinoma in all prostatic lobes and finally metastasizing in different organs at 12 weeks of age [39]. However, the genetic background of the mouse might affect the phenotype. The pattern of the disease progression described in the PTEN mouse model is observed in mice of mixed genetic background. However, when a more homogeneous genetic background (up to eight backcrossing in C57BL/6 strain) was studied, mPIN appeared at 2 month of age and invasive adenocarcinoma at 12 months, nevertheless no metastatic lesions were observed [40]. The deficiency of PAP expression in our mice leads to a similar phenotype than that observed inPTEN-deficient mouse model of prostate cancer both with the same genetic background (C57BL/6). The only difference between these mouse models is that there are no pathological changes in VP lobe of PAP^−/−^ mice.

The ultrastructural studies of PAP^−/−^ mouse prostates showed a high amount of nanovesicles, compatible in size with exosomes. According to this finding, other authors have reported a significant increment of exosomes in plasma of prostate cancer patients compared to healthy donors or benign hyperplasia [14]. In addition, King *et al*. showed that augmented levels of hypoxia inside solid tumors increased the release of exosomes [41]. Flotillin as well as bis-(monoacylglycero)-phosphate (BMP or LBPA) are present in exosomes [42], and previously we have shown that PAP co-localized with flotillin in LNCaP cells and with BMP in human prostate cancer samples [5]. We now confirm the presence of TMPAP inexosomes from TMPAP/LNCaP cells; in addition these exosomes also containedsnapin, flotillin and CD13. The microarray results indicate significant changes in the expression of genes related to the release of neurotransmitters and vesicular traffic in prostates of PAP^−/−^ mice. The interaction between TMPAP and snapin detected in LNCaP cellsisanindication that disturbed exocytosis is involved in the phenotype we observed. The vesicular traffic is an intrinsic factor in the regulation of cell polarity, which is gaining attention as a determinant of tumor development [43]. Recently, exosomes and exosome-like vesicles received increased attention in relation to tumor development and progression. In particular, the release of exosomes containing biologically active molecules, such as microRNAs, DNAs, RNAs and proteins, has been reported to have an impact on cell-cell communication. The above mentioned tumor suppressor PTEN is exported in exosomes while maintaining its phosphatase activity in the recipient cells [12], this could imply that cells which do not express certain protein could obtain it from others.

Our results in addition to our previous knowledge of the presence of PAP in the endosomal-lysosomal pathway [5] highlight a new role for PAP in prostatic vesicular traffic which has not been described before. Considering the topology of TMPAP, this enzyme cannot exert cytosolic acid phosphatase activity and consequently it is not able to dephosphorylate cytosolic tyrosines of epidermal growth factor receptor (EGFR) as it has been previously suggested [7]. Therefore, we assume this is not the pathway leading to the observed prostate adenocarcinoma in the PAP^−/−^ mice.

**Figure 7:**
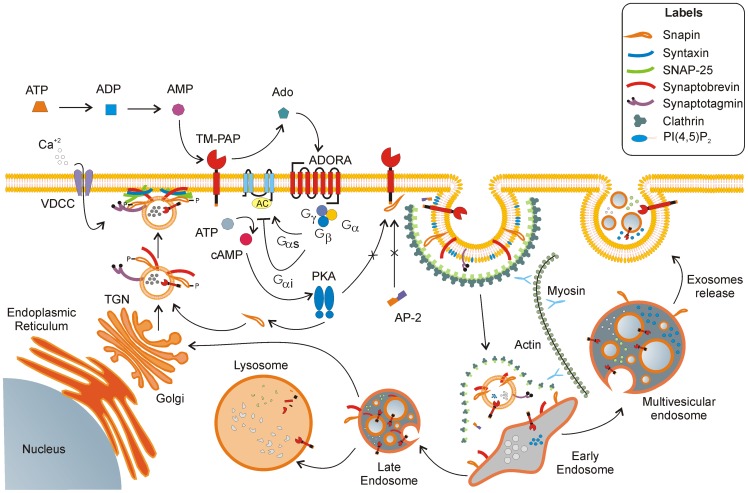
TMPAP is involved in endo-/exocytosis (proposed mechanism). TMPAP synthesized in the endoplasmic reticulum is transported in vesicles to the plasma membrane through the trans-Golgi network (TGN). After the vesicle docking and fusion events leading to release of vesicle content, TMPAP inserted in plasma membrane exerts its phosphatase function over AMP. The resulting product adenosine (Ado) activates the adenosine receptors, which are GPCRs, A1 or A3with G_αi_ (inhibitory G-protein β-subunit) specificity leading to the inhibition of adenylate cyclase (AC) activity, and A2 adenosine receptors with G_αs_ (stimulatory G-protein α-subunit) producing the stimulation of AC activity. Activated AC produces cAMP, which activates PKA responsible for the phosphorylation of snapin. The turnover is completed by clathrin-mediated endocytosis of SNARE components and TMPAP for recycling and degradation in lysosomes vía the endosomal-lysosomal pathway. From early endosomes, the cargo can be sorted to late endosomes or to MVE, which can follow the route leading to exosome release. Additional dephosphorylation events by TMPAP can occur while trafficking between different compartments. From late endosomes, TMPAP can go to lysosomes or back to TGN via the retrograde pathway. ATP: adenosine triphosphate, ADP: adenosine diphosphate, AMP: adenosine monophosphate, Ado: adenosine, TGN: trans-Golgi network, P: phosphate group, AP-2: adaptor protein complex 2, ADORA: adenosine receptor A (types A1, A2 and A3), AC: adenylate cyclase, G_αs_, G_αi_, G_β_, G_γ_: G-protein subunits, VDCC: Voltage-gated calcium channel. Synaptobrevin, syntaxin and SNAP25 are SNARE proteins. PI (4,5) P_2_: phosphatidylinositol 4,5-bisphosphate.

In Figure 7, we summarize our hypothesis about the modulatory effect of TMPAP on endo-/exocytosis and the mechanisms involved in the physical interaction between TMPAP and snapin. Previous reports have showed that the interaction of membrane proteins with snapin negatively regulates exocytosis by affecting the coupling of synaptotagmin to the SNARE complex [44] or by reducing snapin phosphorylation [45] which is needed to strength the interaction between synaptotagmin and Snap25. The phosphorylation of snapin by the cyclic adenosine monophosphate (cAMP)-dependent kinase PKA is crucial step for SNARE assembly in pancreatic β-cells leading to glucose-induced exocytosis [29]. Our results are consistent with these findings, and we built a mechanistic model representing the interaction between TMPAP and snapin. According to Buxton *et al*., 70% of snapin is found in the cytosol [46] where its phosphorylation by PKA occurs [47]. This process would be delayed if snapin is bound to TMPAP and could be a first regulatory effect on secretion. A second effect could involve the 5′-ectonucleotidase activity of TMPAP responsible for the production of adenosine from AMP [19]. Adenosine receptors are G-protein couple receptors (GPCRs) known to regulate neurotransmission/exocytosis [48]. In this case, adenosine could bind to its cognate receptors A1, A2 and A3 that modulate cAMP levels [49], [50] consequently modifying the PKA activity and snapin phosphorylation status. Moreover, the interaction between TMPAP and snapin at the plasma membrane could block the interaction between the cytosolic YxxΦ motif in TMPAP and the adaptor protein complex-2 required for clathrin-based endocytosis [51]. This effect could delay the internalization of TMPAP and extend the time that TMPAP is present in the cellular surface eliciting its catalytic activity and producing a sustained adenosine effect on adenosine receptors.

According to this model, the lack of TMPAP would lead to the observed dysregulation of vesicular traffic, exocytosisand release of exosomes in PAP^−/−^ mouse prostate. This could establish a significant starting point for uncontrolled cell proliferation and the development of prostate adenocarcinoma. Interestingly, in DRG of PAP^−/−^ mice the levels of PI (4,5) P_2_ are increased when compared to wild-type mice [21]. Considering that PI (4,5) P_2_ is the main regulator for clathrin-based endocytosis [20], our observations of increased exocytosis in prostates of PAP^−/−^ mice requires a concomitant increased endocytosis mechanism to keep the cell membrane homeostasis.

In summary, this PAP^−/−^ mouse model shows that TMPAP is required for the normal function of prostate in mice, and its deficiency leads to prostate adenocarcinoma. This suggests that TMPAP acts as a regulator of endo-/exocytosis mechanism.

## Supporting Information

File S1 **Supplementary fileincludes**: Figure S1: Pan cytokeratin immunohistochemistry of DLP from 12 month-old animals. Tables S1: Proliferative cell count statistics. Tables S2: Proliferative and non-proliferative cell counts. Tables S3: Apoptotic cell count statistics. Tables S4: Apoptotic and non-apoptotic cell counts. Tables S5: Significant ontological groups in the cellular component category obtained with Genomatix Bibliosphere software from two-color microarrays experiments. Tables S6: Significant ontological groups in the biological process category obtained with Genomatix Bibliosphere software from two-color microarrays experiments. Supplementarymethodology.
(DOCX)
