# Supplementary material for: Transmembrane Prostatic Acid Phosphatase (TMPAP) Interacts with Snapin and Deficient Mice Develop Prostate Adenocarcinoma
Source: PLoS One. 2013 Sep 10;8(9):e73072. doi: 10.1371/journal.pone.0073072 (PMC3769315; doi:10.1371/journal.pone.0073072)
Supplement: File S1 — Supplementary fileincludes: Figure S1: Pan cytokeratin immunohistochemistry of DLP from 12 month-old animals. Tables S1: Proliferative cell count statistics. Tables S2: Proliferative and non-proliferative cell counts. Tables S3: Apoptotic cell count statistics. Tables S4: Apoptotic and non-apoptotic cell counts. Tables S5: Significant ontological groups in the cellular component category obtained with Genomatix Bibliosphere software from two-color microarrays experiments. Tables S6: Significant ontological groups in the biological process category obtained with Genomatix Bibliosphere software from two-color microarrays experiments. Supplementarymethodology. (DOCX) [file pone.0073072.s001.docx]

**Supplementary material**

Supplementary material comprehends: Figure S1, Tables S1 to S6, and supplementary methods.

**Supplementary figure and tables**

**
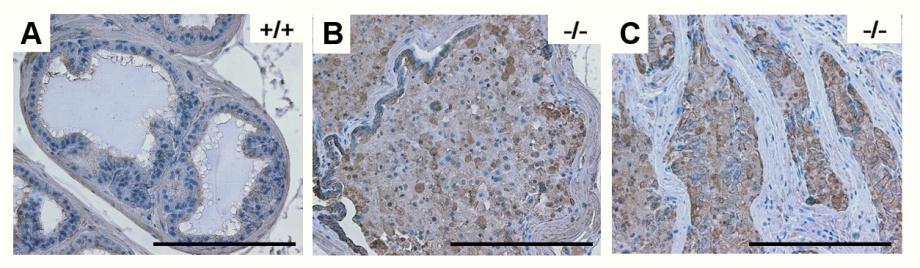
**

**Figure S1** Pan cytokeratin immunohistochemistry of DLP from 12-month-old animals. A, monolayer epithelium with open lumen in PAP^+/+^ mouse. B, epithelial cells crowding into the lumen and stroma of PAP^-/-^ DLP. C, clusters of malignant epithelial cells in the fibrotic stroma. Scale bars: 100 μm (*n*=3).

**Table S1** Proliferative cell count statistics

| Age group (months) | χ^2^ | 95% CI | Proportion 1 | Proportion 2 | P value |
| --- | --- | --- | --- | --- | --- |
| 3 | 8.1548 | 0.0026 – 0.0152 | 0.022 | 0.013 | 0.004 |
| 6 | 63.8851 | 0.0126 – 0.0221 | 0.020 | 0.003 | 1.3 x 10^-15^ |
| 12 | 16.9034 | 0.0063 – 0.0165 | 0.021 | 0.010 | 3.9 x 10^-5^ |

Results for 2-sample test for equality of proportions with continuity correction. Proportion 1: # of proliferative cells in PAP^-/-^ sample / # of total cells in PAP^-/-^ sample; Proportion 2: # of proliferative cells in WT samples / # of total cells in WT sample.

**Table S2** Proliferative and non-proliferative cell counts

| **Phenotype** | **Age**^a^ | **n**^b^ | **Proliferative**^c^ | **Non-proliferative**^d^ | **Total**^e^ |
| --- | --- | --- | --- | --- | --- |
| PAP^-/-^ | 2 | 4 | 76 | 3357 | **3433** |
| PAP^-/-^ | 6 | 4 | 83 | 4037 | **4120** |
| PAP^-/-^ | 12 | 6 | 123 | 5709 | **5832** |
| WT | 2 | 4 | 54 | 4026 | **4080** |
| WT | 6 | 6 | 14 | 5042 | **5056** |
| WT | 12 | 4 | 35 | 3575 | **3610** |

^a^ Age in months

^b^ Number of samples. Ten fields per sample.

^c^ Number of proliferative cells in all the samples

^d^ Number of non-proliferative cells in all the samples

^e^ Total number of cells (proliferative + non-proliferative)

**Table S3** Apoptotic cell count statistics

| Age group (months) | χ^2^ | 95% CI | Proportion 1 | Proportion 2 | P value |
| --- | --- | --- | --- | --- | --- |
| 3 | 1.0714 | -0.0028 – 0.0007 | 0.0003 | 0.001 | 0.30 |
| 6 | 2.3299 | -0.0035 – 0.0005 | 0.0005 | 0.002 | 0.13 |
| 12 | 9.0 x 10^-4^ | -0.0016 – 0.0022 | 0.0016 | 0.001 | 0.97 |

Results for 2-sample test for equality of proportions with continuity correction. Proportion 1: # of apoptotic cells in PAP^-/-^ sample / # of total cells in PAP^-/-^ sample; Proportion 2: # of apoptotic cells in WT samples / # of total cells in WT sample.

**Table S4** Apoptotic and non-apoptotic cell counts

| **Phenotype** | **Age**^a^ | **n**^b^ | **Apoptotic**^c^ | **Non-apoptotic**^d^ | **Total**^e^ |
| --- | --- | --- | --- | --- | --- |
| PAP^-/-^ | 2 | 4 | 1 | 3373 | **3374** |
| PAP^-/-^ | 6 | 4 | 2 | 4221 | **4223** |
| PAP^-/-^ | 12 | 6 | 10 | 6185 | **6195** |
| WT | 2 | 4 | 4 | 2977 | **2981** |
| WT | 6 | 4 | 6 | 3067 | **3073** |
| WT | 12 | 4 | 4 | 2981 | **2985** |

^a^ Age in months

^b^ Number of samples. Ten fields per sample.

^c^ Total number of apoptotic cells

^d^ Total number of non-apoptotic cells

^e^ Total number of cells (apoptotic + non-apoptotic)

**Table S5** Significant ontological groups in the cellular component category obtained with Genomatix Bibliosphere software from two-color microarrays experiments.

| **Term** | **GO ID** | **Total** | **Observed** | **Expected** | **Z-Score** |
| --- | --- | --- | --- | --- | --- |
| neuron projection | GO:0043005 | 104 | 14 | 1.62 | 9.83 |
| axon | GO:0030424 | 59 | 10 | 0.92 | 9.56 |
| extracellular region | GO:0005576 | 1952 | 64 | 30.42 | 6.55 |
| synapse | GO:0045202 | 177 | 13 | 2.76 | 6.25 |
| extracellular space | GO:0005615 | 1763 | 58 | 27.47 | 6.22 |
| cellular component | GO:0005575 | 10650 | 210 | 165.97 | 5.99 |
| extracellular region part | GO:0044421 | 1863 | 59 | 29.03 | 5.97 |
| synapse part | GO:0044456 | 86 | 8 | 1.34 | 5.81 |
| cell projection | GO:0042995 | 275 | 16 | 4.29 | 5.75 |
| integral to membrane | GO:0016021 | 3188 | 80 | 49.68 | 4.85 |
| intrinsic to membrane | GO:0031224 | 3198 | 80 | 49.84 | 4.82 |
| synaptic vesicle | GO:0008021 | 51 | 5 | 0.79 | 4.76 |
| membrane | GO:0016020 | 4484 | 101 | 69.88 | 4.43 |
| membrane part | GO:0044425 | 3536 | 83 | 55.1 | 4.29 |
| clathrin-coated vesicle | GO:0030136 | 83 | 6 | 1.29 | 4.18 |

**Term:** associated ontological term**. GO ID**: gene ontology ID accession number. **Total**: total number of genes annotated to the GO ID entry. **Observed**: number of terms observed. **Expected**: number of terms expected based on observed values. **Z-Score**: for a term indicates how far that term deviates from its distribution’s mean expressed in units of its distribution’s standard deviation. Significant Z-Score > 4.0. **Grayed-rows**: relevant ontological groups for vesicular transport. Mice in microarray experiment per group, n=3.

**Table S6** Significant ontological groups in the biological process category obtained with Genomatix Bibliosphere software from two-color microarrays experiments.

| **Term** | **GO ID** | **Total** | **Observed** | **Expected** | **Z-Score** |
| --- | --- | --- | --- | --- | --- |
| fluid transport | GO:0042044 | 12 | 4 | 0.21 | 8.26 |
| water transport | GO:0006833 | 12 | 4 | 0.21 | 8.26 |
| biological process | GO:0008150 | 10531 | 245 | 187.94 | 7.22 |
| intermediate filament cytoskeleton organization and biogenesis | GO:0045104 | 16 | 4 | 0.29 | 7.02 |
| intermediate filament-based process | GO:0045103 | 19 | 4 | 0.34 | 6.35 |
| synaptic transmission | GO:0007268 | 171 | 13 | 3.05 | 5.78 |
| multicellular organismal process | GO:0032501 | 2504 | 79 | 44.69 | 5.64 |
| transmission of nerve impulse | GO:0019226 | 203 | 14 | 3.62 | 5.54 |
| cell activation | GO:0001775 | 211 | 14 | 3.77 | 5.36 |
| neuron migration | GO:0001764 | 54 | 6 | 0.96 | 5.19 |
| leukocyte activation | GO:0045321 | 197 | 13 | 3.52 | 5.14 |
| regulated secretory pathway | GO:0045055 | 55 | 6 | 0.98 | 5.12 |
| cell-cell signaling | GO:0007267 | 302 | 17 | 5.39 | 5.09 |
| endocrine system development | GO:0035270 | 41 | 5 | 0.73 | 5.04 |
| negative regulation of multicellular organismal process | GO:0051241 | 28 | 4 | 0.5 | 5 |
| neurotransmitter secretion | GO:0007269 | 43 | 5 | 0.77 | 4.88 |
| system development | GO:0048731 | 1480 | 50 | 26.41 | 4.86 |
| axonogenesis | GO:0007409 | 141 | 10 | 2.52 | 4.78 |
| generation of a signal involved in cell-cell signaling | GO:0003001 | 79 | 7 | 1.41 | 4.76 |
| neurological process | GO:0050877 | 449 | 21 | 8.01 | 4.7 |
| nervous system development | GO:0007399 | 547 | 24 | 9.76 | 4.68 |
| forebrain development | GO:0030900 | 82 | 7 | 1.46 | 4.63 |
| neuron morphogenesis during differentiation | GO:0048667 | 149 | 10 | 2.66 | 4.56 |
| neurite morphogenesis | GO:0048812 | 149 | 10 | 2.66 | 4.56 |
| lymphocyte activation | GO:0046649 | 179 | 11 | 3.19 | 4.43 |
| generation of neurons | GO:0048699 | 289 | 15 | 5.16 | 4.41 |
| immune system development | GO:0002520 | 234 | 13 | 4.18 | 4.39 |
| localization | GO:0051179 | 2280 | 66 | 40.69 | 4.33 |
| T cell activation | GO:0042110 | 111 | 8 | 1.98 | 4.33 |
| dorsal/ventral pattern formation | GO:0009953 | 37 | 4 | 0.66 | 4.15 |
| neurogenesis | GO:0022008 | 308 | 15 | 5.5 | 4.13 |
| multicellular organismal development | GO:0007275 | 1896 | 56 | 33.84 | 4.1 |
| cellular morphogenesis during differentiation | GO:0000904 | 169 | 10 | 3.02 | 4.08 |
| regulation of neurotransmitter levels | GO:0001505 | 75 | 6 | 1.34 | 4.08 |
| anatomical structure development | GO:0048856 | 1699 | 51 | 30.32 | 4.01 |

**Term:** associated ontological term**. GO ID**: gene ontology ID accession number. **Total**: total number of genes annotated to the GO ID entry. **Observed**: number of terms observed. **Expected**: number of terms expected based on observed values. **Z-Score**: for a term indicates how far that term deviates from its distribution’s mean expressed in units of its distribution’s standard deviation. Significant Z-Score > 4.0. **Grayed-rows**: relevant ontological groups for vesicular transport. Mice in microarray experiment per group, n=3.

**Supplementary methods**

**Histology**

Mouse prostates were formalin-fixed and embedded in paraffin. Five µm serial sections were stained with hematoxylin and eosin and evaluation of histology was performed following the previously described guidelines [1]. Six to eight mice of each age group were analyzed. Images were taken with Labovert FS microscope (Leica Microsystems GmbH) and Nikon Eclipse E800 with Nikon DS-Ri1 camera and NIS-Elements Basic research Version 4.12 computer program.

**Smooth muscle actin and pan cytokeratin staining**

Five μm sections of paraffin-embedded mouse prostates were deparaffinized, rehydrated and treated for antigen retrieval in microwave oven 10 min. in 0.01 M sodium citrate buffer, pH 6. The smooth muscle actin was detected with mouse monoclonal anti-smooth muscle β-actin antibody (ASM-1, 1:200, Progen Biotechnik), and the epithelial cells were stained with mouse monoclonal anti-pan cytokeratin (1:250, Abcam). Unspecific binding of the antibody was blocked using BEAT™ Blocker Kit (HistoMouse™, Life Technologies). Immunohistochemical detection was performed with Histomouse Max DAB-Kit (Life Technologies). Specimens were counterstained with hematoxylin. Images were taken with Labovert FS microscope (Leica Microsystems GmbH).

# Proliferation and apoptosis analyses

# The cell proliferation and apoptosis indexes were determined for the PAP^-/-^ and PAP^+/+^ DLP lobes from 3, 6 and 12 month old mice, 4 mice per group. Ki67 staining (Abcam) with **Vectastain Elite ABC Kit (**Vector Laboratories) was used to detect the proliferating cells, which counterstained with hematoxylin. Terminal deoxynucleotidyl transferase–mediated dUTP nick end labeling (TUNEL) staining of the apoptotic cells was conducted with FragEL DNA Fragmentation Detection Kit (Calbiochem) following the manufacturer’s instructions. Ki-67- and TUNEL-positive cells were quantified as follows: 5 μm consecutive sections of paraffin-embedded DLP lobes were either Ki-67 or TUNEL stained. Sections were photographed under Labovert FS microscope (magnification 40x), 10 random fields from each section. Each field was counted for proliferative and apoptotic status. The ratio of positive cells to the total amount of cells per sample were compared between PAP^+/+^ and PAP^-/-^ groups using two-sample t-test for equality of proportions with continuity correction for each age group as implemented in R statistical package version 2.10.1.

**Microarray analyses**

The microarray experiments were performed by Biomedicum Functional Genomics Unit (FuGU) at Biomedicum Helsinki.Two-Color Microarray-Based Gene Expression Analysis: Total RNA was isolated from two-month-old PAP^-/-^ and PAP^+/+^ mice prostates (3 mice/group) with RNeasy Midi kit (Qiagen). Prior to total RNA isolation prostates were stored in RNA stabilization reagent (RNAlater, Qiagen). The labeling and hybridization were done according to manufacturer´s instructions. Combined experimental and reference samples were mixed with blocking agent, fragmentation and hybridization buffer, and hybridized to Agilent Mouse Genome Microarray 4x44K for 17 hours at 65°C. Microarrays were washed and scanned with Agilent Scanner, using protocols provided by the manufacturer. Feature Extraction software was used for image analysis. All control spots were excluded. Also saturated spots (either the field ‘gIsSaturated’ or ‘rIsSaturated’ has ‘1’) were excluded. Within-slide normalization using Local Weighted Scatterplot Smoother (LOWESS) was performed using the implementation in Agilent Feature Extraction software. The methodology used to find the genes with expression changes was done as described elsewhere [2].

One-Color Microarray-Based Gene Expression Analysis: Total RNA was isolated from two-month-old mice prostates as described above, 8 mice/group. Total RNA (200 ng) was primed using oligo-dT (T7) promoter primers and converted to cDNA with  AffinityScript RNase Block (mix of enzymes, Agilent). Primed cDNA was amplified and labeled using fluorescently labeled dNTP-nucleotides (Cy3) and T7 RNA Polymerase. Clean-up of labeled and amplified samples was performed by using Qiagen´s RNeasy mini spin columns. RNA Spike-In Kit was used to monitor the success of the labeling. Purified samples were mixed with blocking agent, fragmentation and hybridization buffer, and were hybridized to Agilent SurePrint G3 Mouse GE 8x60K Microarrays for 17 hours at 65°C. Microarrays were washed and scanned with Agilent Scanner using protocols provided by the manufacturer. Feature Extraction software was used for image analysis. For normalization the data were analyzed using the Anduril bioinformatics framework [3]. Probe intensities were normalized with LOWESS using Agilent Feature Extractor. Probes that are not above microarray background signal or whose sequences could not be mapped to Ensembl v61 transcripts were discarded [4]. Probes with median fold change > 1.5 or < 1/1.5 and with FDR-corrected [5] P-value < 0.05 were considered as differentially expressed.

Ontological analyses of microarray results. Results from two-color microarray-based gene expression were analyzed using GoMiner software [6], with significant ontological groups having P-value changed < 0.05, and Genomatix Bibliosphere (Genomatix Software GmbH) with significant ontological groups having co-citation z-score > 4.0. Genes were considered significantly downregulated or upregulated if the strength value was less than -1.8 or greater than 1.8 respectively. Ontological analysis for one-color microarray-based gene expression was made with GoMiner software. Genes were considered significantly downregulated or upregulated if the fold-change was less than -1.5 or greater than 1.5 and the FDR-corrected P-value was less than 0.05.

**Comparative genomic hybridization (CGH)**

The labeling and hybridization were done according to the manufacturer´s instructions. The genomic PAP^-/-^ mouse prostate DNA was purified from two 6-month-old, two 15-month-old and one 25-month-old mice with DNeasy Blood & Tissue Kit (Qiagen) according manufacturer’s instructions. The control DNA was purified similarly from four two-month-old wild-type mouse prostates and pooled. The starting amount of genomic DNA was 1μg. A total of 1μg of labeled samples was used for hybridization. Agilent Oligonucleotide Array-Based CGH for Genomic DNA Analysis was used for PAP^-/-^ mice as follows: 1μg of genomic DNA was digested with Alu I and Rsa I enzymes.  Digested genomic DNA samples were labeled using random primers, fluorescent-labeled dUTP nucleotides (Cy3 and Cy5) and the exo-Klenow fragment. Clean-up of labeled genomic DNA was performed by using Microcon YM-30 filters. Combined experimental and reference samples were mixed with Cot-1 DNA, blocking agent and hybridization buffer, and hybridized to Agilent Mouse CGH 4 x 180K Oligo Microarrays for 24 hours at 65°C. Microarrays were washed and scanned with Agilent Scanner, using protocols provided by the manufacturer. Feature Extraction software was used for image analysis.

**Generation of stable transfected LNCaP cells**

Human TMPAP cDNA was cloned between BamHI-NotI in pMX neo vector [7]. The human Phoenix gag-pol packaging cell line was cultured in high-glucose DMEM containing 10 % FCS, 2 mM glutamine, and 100 U/ml penicillin and 100 µg/ml streptomycin (Sigma). Confluent cells in six-well plates were transfected with 4 µg of pMX neo-TMPAP or pMX neo and 0.4 µg of pVSV-G retroviral vector per well by using Lipofectamine 2000 (Invitrogen) according to the manufacturer's instructions. Twenty-four hours post-transfection, the medium was changed to low-glucose DMEM with the same supplements as above. Forty-eight hours post-transfection, the viral media was collected every 24h for up to 6 days. LNCaP cells (ATCC) were cultured in RPMI-1640 with 10 % FCS, 2 mM glutamine, and 100 U/ml penicillin and 100 µg/ml streptomycin (Sigma) in six-well plates. When cells were 70 % confluent the medium was changed to 1 ml of virus-containing (TMPAP pMX or empty pMX virus) supernatant with 4 µg/ml polybrene (Sigma) per well and incubated at 37^o^C. Twenty-four hours after the first infection, the medium was removed and cells were infected again as above. Forty-eight hours after the first infection, cells were trypsinized and seeded on a six-well plate in presence of 400 µg/ml geneticin (Roche) for selection. Antibiotic selection was continued until cells on the control wells have died. TMPAP-transfected and pMX-transfected LNCaP cells (TMPAP/LNCaP and pMX/LNCaP cells) were grown and maintained in RPMI-1640 with 10 % FCS, 2 mM glutamine, and 100 U/ml penicillin, 100 μg/ml streptomycin and 200 µg/ml geneticin. TMPAP production was assayed by quantitative RT-PCR or immunoblotting.

**Immunofluorescence and co-localization studies**

TMPAP/LNCaP and pMX/LNCaP cells were seeded on 12-mm glass coverslips (BD Biosciences) and fixed with 4% paraformaldehyde followed by 5 min. methanol at -20ºC, and blocked using 0.2% Saponin, 5% fat free dry milk in PBS for 30min. Double immunostaining: cells were incubated overnight at 4ºC with primary mouse monoclonal anti-human PAP (1:100, Sigma) and rabbit polyclonal anti-snapin (1:200, Synaptic System) antibodies. Cells were then incubated with Alexa Fluor 488 goat anti-mouse, and Alexa Fluor 594 goat anti-rabbit (Invitrogen) antibodies for one hour at RT. The samples were mounted (Mowiol/DABCO/DAPI), and confocal images were acquired using an Olympus FluoView 1000 confocal microscope with UPLSAPO 60x oil NA 1.35 objective, and 405, 488, and 543 nm laser excitation. Three different experiments were performed and three different fields were analyzed per sample. Seven to thirteen stack images were acquired for each field using sequential scanning, with an image size of 1024x1024 and 500 nm in z-dimension.

Confocal stack images were deconvolved with 5 iterations using AutoQuantX's blind deconvolution algorithm (Media Cybernetics). The 3D co-localization studies were performed using Biplane Imaris 7.2 software (Biplane AG) and the automatic threshold co-localization algorithms developed by Costes et. al. [8]. The co-localization results are expressed as a mean of Pearson’s coefficient ± SEM.

**Isolation of exosomes and Western blot**

TMPAP/LNCaP cells were seed and allowed to attach in RPMI-1640 complete medium (10 % FCS, 2 mM glutamine, 100 U/ml penicillin, 100 µg/ml streptomycin and 200 µg/ml geneticin) for 24 hs. Cells were washed thoroughly with PBS and cultured for 72 hs in RPMI1640 serum free medium (2mM glutamine, 100 U/ml penicillin, 100 µg/ml streptomycin and 200 µg/ml geneticin). The medium was recovered for the isolation of exosomes [9]. Consecutive centrifugations 5 min. at 500 g and 15 min. at 2000 g were performed to remove cellular debris. Supernatants were then filtered through out a 0.22 µm filter to remove any large vesicles. The culture media were then concentrated as maximum 35 times using AMICON concentrators (10 kDa cut off). The concentrated media were ultracentifuged for 2 h 100000 x g (Beckman Optima LE-80K, 50Ti rotor), the pellets were washed in 1ml PBS, and ultracentrifuged again for 2 h at 100,000 g. Finally, the pellets containing the exosomes were resuspended in 30 µl PBS and store at -70ºC for further analysis. Protein concentrations were measured with Pierce BCA Protein Assay kit (Thermo) following manufacturer’s instructions. Samples were loaded in 7.5 % or 12 % ready-made SDS-PAGE gel (BioRad) and blotted into PVDF membrane. Flotillin (BD, 1:500), CD13 (Abcam, 1:1000), PAP (Sigma, 1:800) and snapin (Synaptic Systems, 1:500) were tested. Protein detection was performed with Chemiluminescent substrate (Super Signal West Pico, Thermo) following the manufacturer’s instructions.

**References**

1. Shappell SB, Thomas GV, Roberts RL, Herbert R, Ittmann MM, et al. (2004) Prostate pathology of genetically engineered mice: Definitions and classification. the consensus report from the bar harbor meeting of the mouse models of human cancer consortium prostate pathology committee. Cancer Res 64: 2270-2305.

2. Kauraniemi P, Hautaniemi S, Autio R, Astola J, Monni O, et al. (2004) Effects of herceptin treatment on global gene expression patterns in HER2-amplified and nonamplified breast cancer cell lines. Oncogene 23: 1010-1013. 10.1038/sj.onc.1207200.

3. Ovaska K, Laakso M, Haapa-Paananen S, Louhimo R, Chen P, et al. (2010) Large-scale data integration framework provides a comprehensive view on glioblastoma multiforme. Genome Med 2: 65. 10.1186/gm186.

4. Gertz EM, Sengupta K, Difilippantonio MJ, Ried T, Schaffer AA. (2009) Evaluating annotations of an agilent expression chip suggests that many features cannot be interpreted. BMC Genomics 10: 566. 10.1186/1471-2164-10-566.

5. Benjamini Y, Hochberg Y. (1995) Controlling the false discovery rate: A practical and powerful approach to multiple testing. J R Statist Soc 57: 289-300.

6. Zeeberg BR, Feng W, Wang G, Wang MD, Fojo AT, et al. (2003) GoMiner: A resource for biological interpretation of genomic and proteomic data. Genome Biol 4: R28.

7. Kitamura T. (1998) New experimental approaches in retrovirus-mediated expression screening. Int J Hematol 67: 351-359.

8. Costes SV, Daelemans D, Cho EH, Dobbin Z, Pavlakis G, et al. (2004) Automatic and quantitative measurement of protein-protein colocalization in live cells. Biophys J 86: 3993-4003. 10.1529/biophysj.103.038422.

9. Tauro BJ, Greening DW, Mathias RA, Ji H, Mathivanan S, et al. (2012) Comparison of ultracentrifugation, density gradient separation, and immunoaffinity capture methods for isolating human colon cancer cell line LIM1863-derived exosomes. Methods 56: 293-304. 10.1016/j.ymeth.2012.01.002; 10.1016/j.ymeth.2012.01.002.
